# Utilization of aquatic biomass as biosorbent for sustainable production of high surface area, nano- microporous, for removing two dyes from wastewater

**DOI:** 10.1038/s41598-024-54539-2

**Published:** 2024-02-23

**Authors:** Maha Ahmed Mohamed Abdallah, Ahmed E. Alprol

**Affiliations:** National Institute of Oceanography and Fisheries, NIOF, Cairo, Egypt

**Keywords:** Removal, Congo red, Xylenol orange, Wastewater, Biomass, Isotherm models, Kinetic models, Environmental sciences, Nanoscience and technology

## Abstract

The majority of environmental researchers are becoming increasingly concerned with the manufacture of inexpensive adsorbents for the detoxification of industrial effluents. To address one of the significant and well-known pollution issues with certain drains that act as hotspots and contribute to coastal pollution in Alexandria, this study aims to develop an economical, ecologically friendly sorbent. This study assessed the efficacy of a biomass-coated magnetic composite and a magnetic active adsorbent for the removal of two dyes from an industrially contaminated sewer using a wetland plant (*Phragmites australis*). Using magnetic biosorbent, the biosorption of Xylenol orange and Congo red ions from polluted drain discharge in Abu Qir Bay was evaluated in the current study. Using scanning electron microscopy imaging and Fourier transform infra-red analysis; the surface function and morphology of the nano-biosorbent were examined. At room temperature, the effects of initial dye concentration, pH, contact time, and nano-biosorbent concentration have all been investigated. The greatest percentages that nano-biosorbent can remove from Congo red and Xylenol orange are 97% and 47%, respectively. The removal of the initial Congo red concentration varied from 42 to 97%, while the removal of the initial Xylenol orange concentration varied from 30 to 47%. The adsorption capacity was shown to be strongly pH-dependent; capacity dose as pH value increased, with pH 10 being the ideal pH for Congo red and pH 6 being the ideal pH value for Xylenol orange. The adsorption capacity for Congo red varied between 0.96 and 3.36 and the adsorption capacity for Xylenol orange varied between 0.18 and 17.58. The removal capacity decreased from 3.36 to 0.96 mg/g when the biosorbent dosage was increased from 0.05 to 0.5 g/L for Congo red, in case of Xylenol orange, the removal capacity increased from 0.18 to 17.58 mg/g when the biosorbent dosage was increased from 0.05 to 0.5 g/L. The removal capacity of Congo red increases quickly with time and varied from 1.66 to 1.88 of contact time; while the removal capacity of Xylenol orange varied between 3.08 and 4.62 of contact time. For the dyes under study, kinetics and adsorption equilibrium were examined. Within 180 min, the equilibrium was attained because to the quick adsorption process. For Congo red and Xylenol orange, the highest adsorption capacities were 3.36 and 17.58 mg g^−1^, respectively. The equilibrium data were assessed using a number of isotherm models, including Langmuir, Freundlich, BET, and Tempkin, while the kinetic data were examined using a variety of kinetic models, including pseudo-first- and pseudo-second-order equations. The pseudo-second-order equation provides the greatest accuracy for the kinetic data and Langmuir model is the closest fit for the equilibrium data.

## Introduction

The public is very concerned about organic and inorganic chemical pollution of water. Water sources are either directly or indirectly exposed to the hazardous effluents from the textile and other industries. These days, adsorption technique is widely employed to remove these kinds of contaminants from wastewaters^1,2^. Dyes and pigments are widely used in a variety of industrial processes, including the production of colored goods for the paper, plastic, leather, textile, and cosmetic industries^3^. Due to their recalcitrance and visibility even at very low concentrations, synthetic dyes, especially organic dyes, are found in many industrial effluents, particularly in the textile industries. These dyes have negative effects on the environment and, consequently, on living things. Since traditional treatment procedures do not work, these organic contaminants are released into the aquatic environment as colored and contaminated waters.

**Figure 1:**
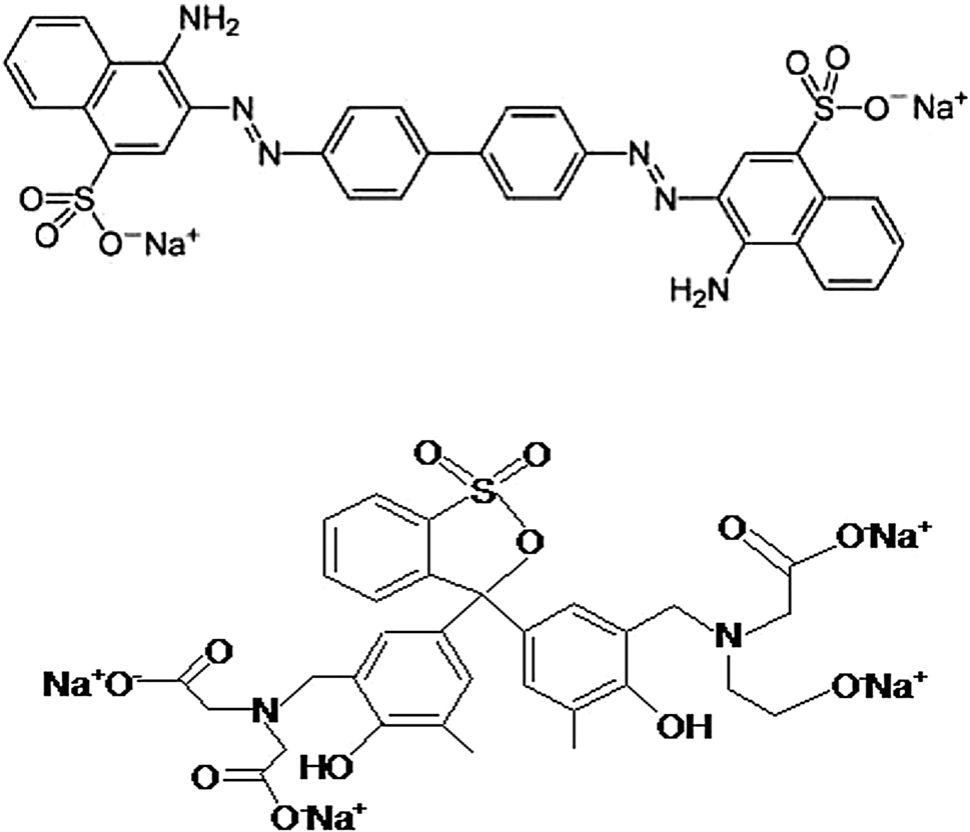
Structure of Congo red and Xylenol orange.

Due to their toxicity and carcinogenicity, as well as the fact that they can cause allergic dermatitis, skin irritation, cancer, and human mutation, colored contaminated effluents are regarded as one of the most important environmental problems in the world, particularly in developing nations^4–6^. Congo red (CR) is an anionic dye [1-naphthalene sulfonic acid, 3,3′-(4,4′-biphenylenebis (azo)) bis(4-amino-) disodium salt, MW = 696.665] and a diazo dye and is obtained by coupling tetrazotised benzidine with two molecules of napthionic acid, forming a structure as shown in Fig. 1. One of the hazardous dyes (human carcinogens) present in wastewater is an anionic diazo dye based on benzidine that has a greater solubility in water of roughly 1 g/30 mL^7^. Several businesses, including the textile, paper, plastic, printing, and dyeing sectors, produce Congo red. In addition to its toxicity and negative impacts on the environment, Congo red's removal from wastewater samples is crucial because of its complex aromatic structure, toxicity to a wide range of organisms, potential carcinogenicity, and mutagen dye status^8^.

The heterocyclic species Xylenol orange, or [3,3-bis-N, N, Di-(carboxymethyl) aminomethyl-O-cresolsulfone-phthalein tetrasodium salt] (Fig. 1), is a member of the acidic dye family. It is an effective potentiometric reagent that is utilized to measure a variety of metal ions. When Xylenol orange is present in the aquatic environment, it attracts heavy metals that can lead to a variety of illnesses in aquatic species and humans. Dyes have been extracted from colored water using a variety of physical, chemical, and microbiological techniques, such as membrane separation, ion exchange, biological degradation, electrochemical methods, and adsorption. Since it is non-toxic, has a high sorption capacity, and is readily available in a variety of sorbent forms, adsorption is currently one of the most effective methods for treating organic pollutants. Adsorption onto nanoparticles has been widely employed as an adsorbent for the removal of organic pollutants due to its high surface area and high uptake capacity^9^.

Lately, the application of inexpensive adsorbents has received increased attention, Dakhil et al.^10^ conducted research on chemically modified rice husks (MRH) for the purpose of reducing aniline ions from industrial wastewater. Dakhil^11^ Investigate if agricultural wastes, such as rice husk (RH), sawdust (SD), and date palm fibers (DPF), may be used as inexpensive adsorbents to remove methyl orange dye (MO) from industrial effluent. He demonstrated that agricultural wastes are an excellent way to purify industrial effluent that includes MO dye.

One of the most extensively found flowering plants in Egypt is *Phragmites australis*, which can be found in large quantities in all the Northern Delta brackish lakes in Egypt^12^. Compared to native strains, the species of grass tends to emerge earlier and is less vulnerable to insect herbivory. It is also harmful to native plants and wildlife and is difficult to eradicate.

Due to the small size of Fe_3_O_4_ nano-sorbents, which is advantageous for the diffusion of pollutants from the solution onto the active sites of the adsorbent surface, nano-magnetic iron oxide has demonstrated a high potential for pollutant uptake in recent studies. When it comes to extracting nanoparticles from aqueous solutions, magnetic separation is thought to be a quick and efficient method when compared to centrifugation and filtration. In this work, we produced a biomass-coated magnetic composite and magnetic active adsorbent (*Phragmites australis*) and assessed their efficacy in removing two dyes from the industrial wastewater from the contaminated drain. In this study, the surface functional groups of the biosorbent under investigation were characterized through FT-IR analysis, which was used to investigate the processes and mechanisms of the dye-sorbent interaction. The goal of this project is to create an affordable, environmentally friendly sorbent to address one of the major and well-known pollution issues with certain drains that serve as hotspots and contribute to coastal pollution in Alexandria.

## Materials and methods

**Figure 2:**
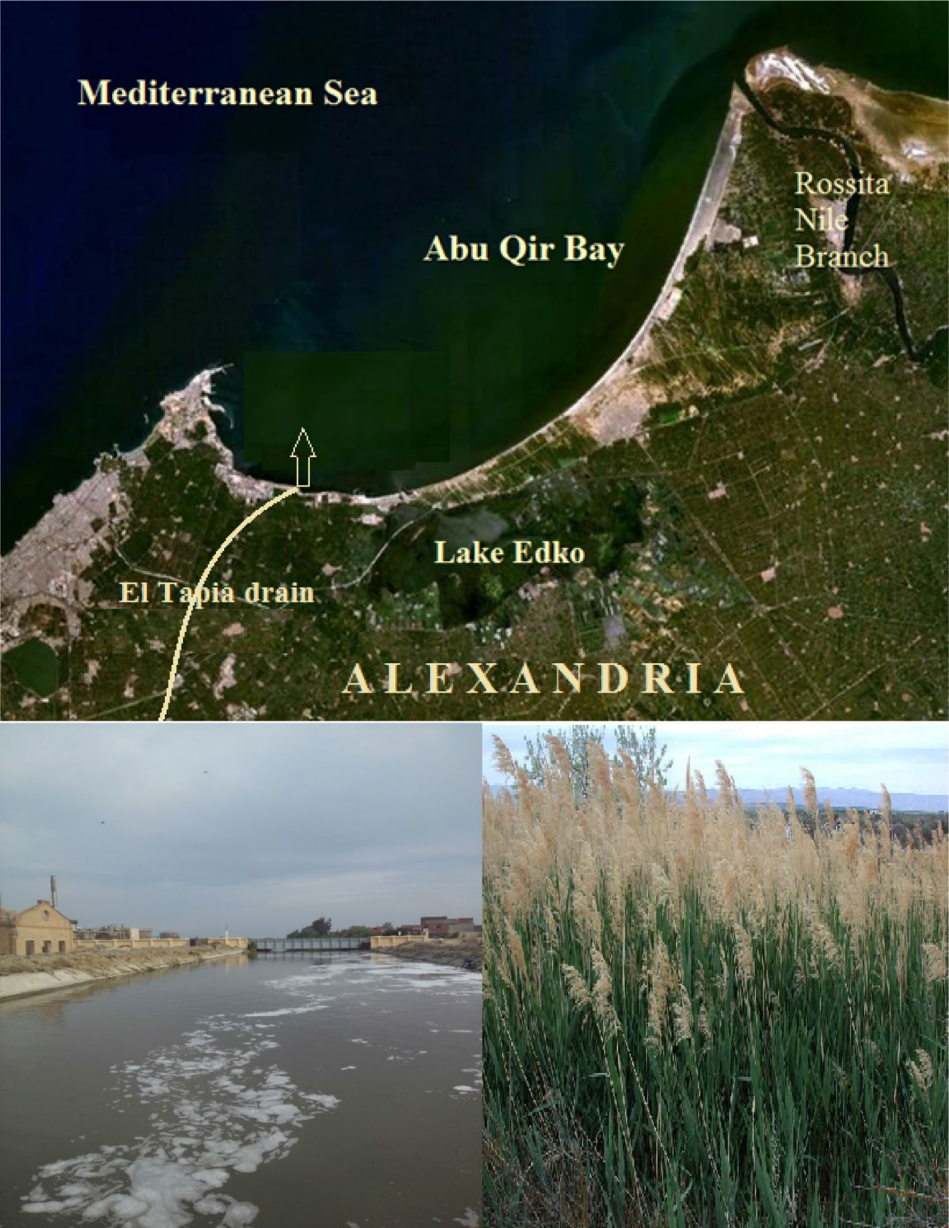
Study area of El Tapia drain (industrial wastewater) in Abu Qir Bay and biomass *(phragmites australis)* [Data SIO, NOAA, US. Navy, NGA, GEBCO Image Landsat/Copernicus Image IBCAO].

Abu Qir Bay receives about 2 × 10^6^ m^3^/day of a mixture of untreated sewage and industrial wastes discharged from Abu Qir drain and dumped into the Bay through the ‘‘Tabia pump station’’; these industrial wastes include fertilizers, textiles, chemicals, dyes, food processing and canning wastes as well as mill paper effluents (about 101,850 m^3^/day). The industrial wastewaters for the present study were collected from this drain (Fig. 2).

### Synthesis of Fe_3_O_4_ nano-sorbents -immobilized macrophytes sorbents

Monometallic catalysts are gold nanoparticles, silver nanoparticles, ZnO nanoparticles, copper nanoparticles, nickel nanoparticles, iron nanoparticles^13^ and platinum nanoparticles^14^. Amounts of the biomass (*Phragmites australis,* Fig. 2) were collected from nearby wetlands around Alexandria, washed with abundant potable water in order to eliminate solid particles, and then air-dried at room temperature. Finally, the washed air-dried macrophytes were dried in the oven at 65 °C for 24 h, crushed, and sieved through a 0.5-mm sieve. Crushed materials were then stoRed at room temperature in an airtight pack and used for further dye removal experiments^9,14^.

Magnetic active adsorbent and the powdered biomass coated magnetic composite were synthesized. In this study, a method was established and presented to enhance and improve the adsorptive efficiency of Fe_3_O_4_ nano-sorbents (modified nanoparticles) for the pre-concentration of some dyes from industrial wastewater by simple, direct and affordable physical treatment of macrophytes, as a source of chelating functional groups. The bio-sorbent interaction processes and mechanisms were investigated in this study using FT-IR analysis, which characterized the surface functional groups of the studied nano magnetite-biosorbent^9^.

### Synthesis of dyes

The dye, Congo red (CR) is the sodium salt of benzidinediazo-bis-1-naphthylamine-4-sulfonic acid having a chemical formula C_3_2H_2_2N_6_Na_2_O_6_S_2_: and molecular weight: 696.66 g/mol, and Xylenol orange (XO) is a tetra-sodium salt having a chemical formula C_31_H_28_N_2_Na_4_O_13_S: and molecular weight 760.58 g/mol, used in this study was supplied by Sigma–Aldrich, India. A spiking standard of CR and XO were prepared by dissolving an accurately weighed quantity of separate dyes in double distilled water to prepare the stock solutions (500 mg/L) of the two dyes separately and employed in the preparation of working standards. All working samples of wastewater were spiked with both studied dyes that were separately prepared. One strip of drain wastewater was analyzed without additional dyes. Serial spiking dilutions were made by diluting two dyes with industrial wastewater^15,16^.

### Biosorption studies

The experimental solution was determined by diluting the dye stock solution inaccurate volume of industrial wastewater needed for initial concentrations. The removal experiments were conducted with parameters such as dye’s initial concentration was estimated by contacting 0.1 g of nano-biosorbent with 100 mL of dye’s solutions of different initial concentrations ranging from 5 to 100 mg/L for CR and from 5 to 60 mg/L for XO and agitation speed of 200 rpm were maintained^4^. The effect of the initial pH on the adsorption of the two dyes adsorbed was obtained by agitating 0.1 g of Fe_3_O_4_ nano-sorbents in a series of bottles containing 100 mL of spiked wastewater solution of initial concentration 60 mg/L for CR and 35 mg/L for XO at different pH from 2.0 to 10.0 by using diluted solutions of HCl and NaOH. The effect of the amount of nano-biosorbent used on the equilibrium uptake was estimated by agitating the dyes spiked wastewater solution of optimum initial concentrations, with the weighed amounts of nano- biosorbent ranging from 0.05 to 0.35 g. The effect of time on the adsorption of the two dyes was obtained by agitating 0.1 g of the nano-biosorbent with the same volume of spiked wastewater solution (100 ml) at different times from 30 t0 180 min with temperature was maintained at 25 °C agitation speed of 200 rpm. Samples pipetted out at different time intervals were filtered through a 0.45-mm-pore-size cellulose acetate membrane filter and then analyzed for the concentration of the dyes. The concentrations of CR and XO were measuRed on a UV–vis spectrophotometer (ELICO SL 164 Double Beam UV–vis spectrophotometer) at ʎmax = 497 and 575 nm respectively^15^.

The amount of dyes adsorbed onto the nano-biosorbent at equilibrium was calculated from the mass balance of Eq. (1) as given below:

1 $${\text{qe}}=\left({{\text{C}}}_{0}- {{\text{C}}}_{{\text{e}}}\right)\frac{{\text{V}}}{{\text{W}}}$$

where C_0_ and C_e_ are the initial and equilibrium concentrations of dyes (mg/L), respectively, qe is equilibrium dyes concentration on nano-biosorbent (mg/L), V is the volume of the dyes solution (in liters), and W is the mass of the nano-biosorbent used (in grams)^14^.

The surface functionality of sorbent is characterized by its responsibility for all activity and capability for all adsorption properties and processes. In the present study, infrared spectroscopy was used to obtain information about the chemical structure and functional groups of the biosorbent before and after adsorption. The FTIR spectrum was recorded using a Shimadzu Fourier Transform infrared spectrophotometer (FTIR system-BX 0.8009) in the range 500–4000 cm^−1^ to acquire the FT-IR spectra of biosorbent. This biosorbent was also imaged by the use of a scanning electron microscope (JSM-5300, JEOL Ltd.). An ion sputtering coating device (JEOL-JFC-1100E) was used to coat the SEM specimens with gold to increase the conductivity^14^.

### Consent to participate

All authors agree to participate.

### Plant guideline statement

All methods were carried out following relevant guidelines.

## Results and discussion

### Surface characterization of the prepared nano-biosorbent

The tested nano-biosorbent system's surface functional groups were identified and described using Fourier transform infrared (FT-IR) analysis. The different functional groups of nano magnetite-biosorbent are identified using this technique, which also provides chemical and biological details about the surface activity of the material. The primary characteristics of infrared peaks are determined and linked to the characteristics of the biosorbent under study^16^.

The adsorption process within the 400–4000 cm^–1^ wavenumber range was attributed to the nano magnetite–biosorbent FT-IR spectrum by IR Prestige-21, Shimadzu, Japan. The spectra of the investigated nano-biosorbent were attributed to –OH and N–H groups from cellulose, lignin, and water^16^. These spectra showed indications of a mini broadband between 3433.41 and 3460.41 cm^−1^ (before and after adsorption of the two studied dyes). The peak at 1635.68 cm^−1^ for raw nano-biosorbent shifted to 1629.9 and 1639.55 cm^−1^ (after adsorption for CR and XO, respectively), corresponded to C=O and C=C stretching, which may be related to the presence of lignin aromatic bond^17^. The band observed at 2366 cm^−1^ (after adsorption of CR) is assigned to C–H bonds. The presence of C–O is visible at 1406.15–1429.3 cm^−1^ both before and after adsorption^18^. The adsorption of organic molecules from aqueous solution by porous^19^ carbon also depends on the surface functional groups, particularly those that contain oxygen.

Additionally, after XO ions were adsorbed into the adsorbent, the band at 1099.48 cm^−1^ that was assigned to the C–O of alcohol and carboxylic acids^20^ and C–N was shifted to 1055.10 cm^−1^ in the case of CR loaded adsorbent, and to 1030.02 cm^−1^ in other cases. In the current study, peaks between 588 and 584 cm^−1^ were observed before and after adsorption by CR. In the case of XO, this peak disappeared, indicating that additional degradation occurred after adsorption^21^ states that the Fe–O band can exist in the range of 450–600 cm^−1^.

**Figure 3:**
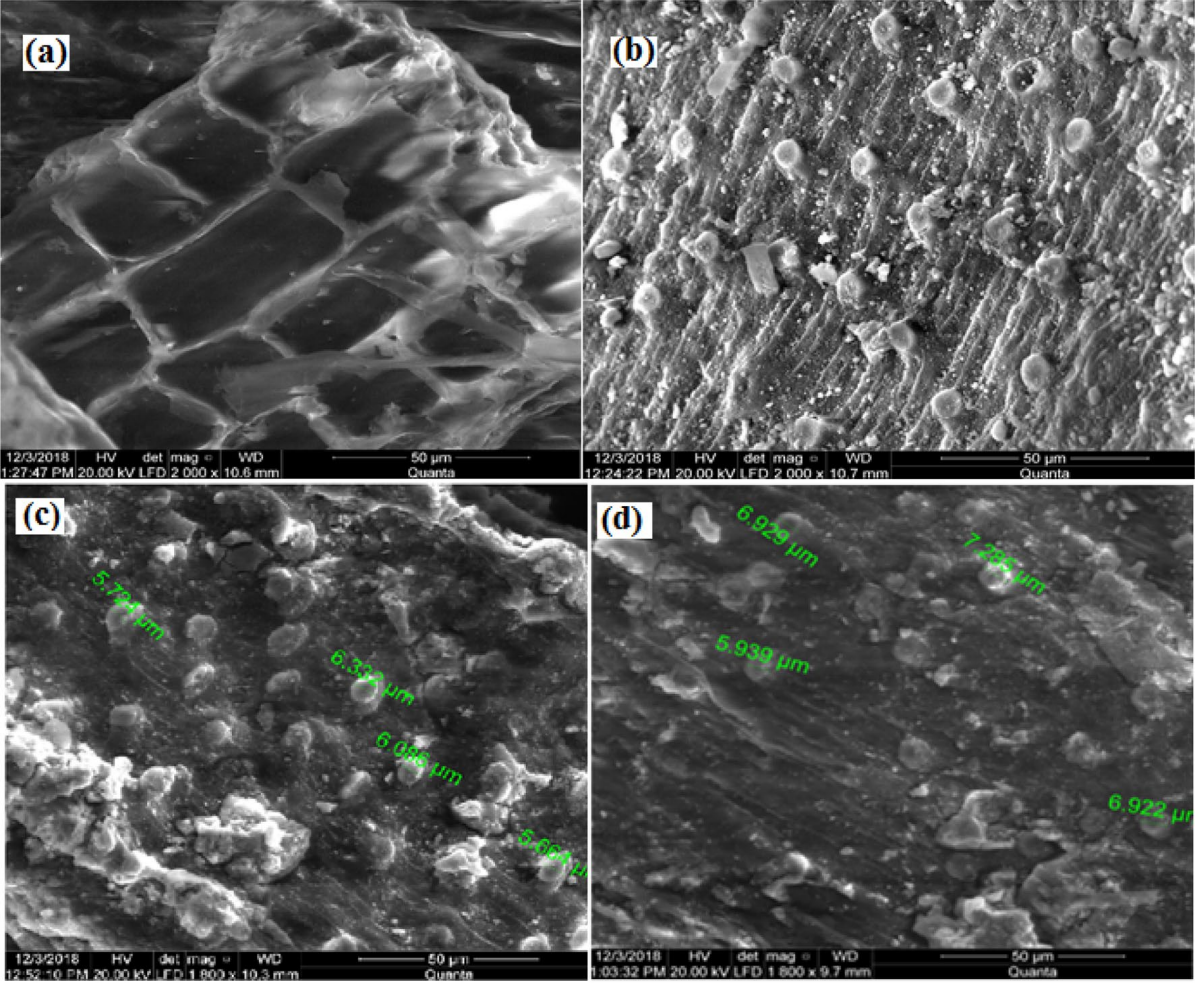
Scanning Electron Microscope of biosorbent before (**a**) and after treatment (**b**) by nano magnetic particles also after adsorption by Congo red (**c**) and Xylenol orange (**d**).

According to FTIR analysis, the interaction of dye ions with adsorbent active sites may be the cause of new absorption bands, changes in absorption intensity, shifts in the wavenumber of functional groups in the nanomagnetic biosorbent, and minor variations in frequency bands. The analyzed nano-biosorbent's spectra, which showed signs of a mini broadband between 3433.41 and 3460.41 cm^−1^ (both before and after the two dyes were adsorbed), were attributed to the –OH and N–H groups found in cellulose, lignin, and water^16^ (Fig. 3).

The band observed at 2366 cm^−1^ (after adsorption of CR) is assigned to C-H bonds and the peak at 1635.68 cm^−1^ for raw nano-biosorbent shifted to 1629.9 and 1639.55 cm^−1^ (after adsorption for CR and XO, respectively), corresponded to C=O and C=C stretching which might be attributed to the presence of lignin aromatic bond^17^. Another peek at 1406.15–1429.3 cm^−1^ before and after adsorption reveals the presence of C–O^17^. Also, the band at 1099.48 cm^−1^ was assigned to the C–O of alcohol and carboxylic acids^20^.

The surface morphology of sorbents can be effectively investigated and evaluated using scanning electron microscopy (SEM). The biosorbents SEM image before and after treatment with nanomagnetic particles is shown in Fig. 2. The natural plant Phragmites australis's surface morphology, which functions as a biosorbent, has maintained its original shape (Fig. 3). However, after being exposed to magnetic nanoparticles, the plant's distribution changed noticeably, exhibiting a large number of active sites. According to additional data, the chemically modified biosorbent underwent noticeable surface alterations after being treated with magnetic nanoparticles, as shown in Fig. 2. In addition, the new biosorbent of active carbon treated by immobilized baker's yeast (4.7455 µm) reported by^9^ has a much richer porous structure, but its mean pore diameter (7.081 µm) is slightly larger. As demonstrated in Fig. 3, the uptake of dyes onto the nanomagnetic biosorbent resulted in the formation of a smoother configuration, regular surface, and expanded pores following the adsorption of CR and XO. The uptake of CR and XO onto the sorbent surface that filled these pores may have caused the pores to expand.

### Removal of dyes from wastewater samples by modified nanoparticles (nano-biosorbent)

#### Effect of initial dye concentration

**Figure 4:**
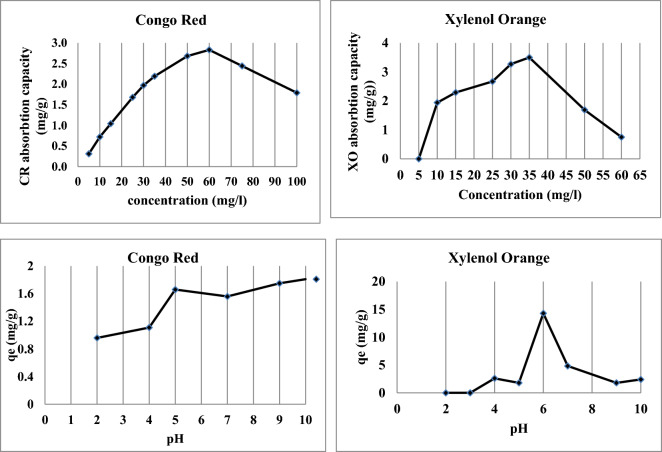
Biosorption of dyes at various concentrations and pH on nano-biosorbent particles.

The removal of dissolved dyes from industrial wastewater through biosorption is a highly efficient process that is meticulously planned and executed. The findings showed that the initial dye concentrations influenced the equilibrium sorption capacity of the nano-biosorbent for dyes, which varied (Fig. 4). As illustrated in Fig. 3, when CR and XO concentrations were raised from 5 to 60 mg/L and from 5 to 35 mg/L, respectively, the biosorption capacity increased from 0.3 to 2.8 and from 0 to 3.5 mg/g. It might be because of the variations in the dye molecules, which raised the likelihood of the dye molecules and the nano-biosorbent colliding, which in turn improved the absorption process. However, the biosorption capacity was reduced at CR concentrations above 60 and XO concentrations above 35 mg/L. When it was discovered that CR and XO had sorption capacities lower than 60 and 35 mg/L, respectively, this could have indicated that the dyes had an inhibitory effect on the nano-biomass, whose cell walls and/or other cellular components served as chemical binding sites. This could, however, be because there aren't enough active sites available at high dye concentrations, increasing competition for available adsorption sites and slowing down the adsorption process.

#### Effect of pH

Adsorption capacity, which influences the adsorbent's surface charge and the extent of ionization of the adsorbate, is largely dependent on pH^20^. Under constant ideal conditions, the extent of removal by nano-biosorbent was investigated at different pH values between pH 2 and pH 10. Regardless of the starting CR concentration, the nano-biosorbent's maximum equilibrium sorption capacity for CR was found at pH 10.0, and for XO, it was found at pH 6.0 (Fig. 4). For CR, it was found that the uptakes were significantly higher in an alkaline solution, and for XO, they were slightly acidic. When the pH was raised from 2 to 6, a decrease in the capacity for XO biosorption was seen. This resulted from how pH affected XO ionization as well as the surface charge of the nano-biosorbent. The number of positively charged surface sites increased and the number of negatively charged surface sites decreased as the system's pH dropped, which promoted the adsorption of dye anions through electrostatic attraction^21^. As the pH of the solution rises, the CR's adsorption increases. This may be explained by the fact that amine groups in the beads readily undergo protonation in low pH (acidic solution) environments^22^.

As a result, protons and the CR competed for adsorption sites, resulting in a drop in adsorption capacity. The amino group was released from protonation for the adsorption behavior in the CR when the pH value rose.

#### Effect of dose

**Figure 5:**
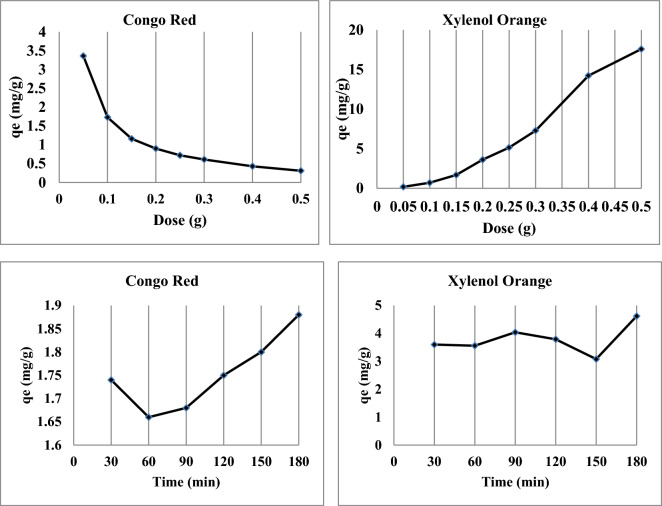
Biosorption capacity of studied dyes at various doses and the effect of contact time of nano-biosorbent particles.

The removal capacity decreased from 3.5 to 0.5 mg/g when the biosorbent dosage was increased from 0.05 to 0.5 g/L, indicating the impact of various adsorbent dosages of nano-biosorbent at an initial CR concentration (100 mg/L) at 25 °C (Fig. 5). A reduction in the sorption amounts was noted in the higher dosages. It might be because the CR molecules are inaccessible and unable to cover all of the adsorbent's surface sites. Additional evidence shows that at higher dosages, a large number of active sites on the surface of the nano-biosorbent are unable to reach a saturation state. When designing sorption systems, adsorption isotherms offer useful specifications for determining the uptake mechanism and sorbent surface properties^23^.

Figure 5 illustrates how different adsorbent (nano-biosorbent) dosages affect the amount of XO removed from industrial effluent. The data indicate that a possible explanation for the observed increase in removal capacity with increasing adsorbent dose is the presence of more adsorption sites and a larger surface area.

#### Effect of time

In Fig. 4, the biosorption of the investigated dyes onto modified nano-biosorbent is shown against contact time at 30, 60, 90, 120, 150, and 180 min. Studies on biosorption were conducted at a concentration of 100 mg/L at 25 °C. The results showed that the removal capacity of CR increases quickly, reaching 1.88 mg/g after 180 min of contact time; in other words, the amount of CR dye adsorbed increased as the reaction time rose.

The initial clearance of XO dye was minimal (first 2.5 h, Fig. 5) and subsequently increased until equilibrium was reached after 3 h (almost total dye removal). This kind of behavior is typical of the biosorbent surface having several adsorption sites during the early stages of the reaction, which gradually become saturated with the dye as contact periods increase.

### Realization of adsorption isotherms

Basic prerequisites for designing biosorption systems that remove organic contaminants from the environment are equilibrium data also referred to as adsorption isotherms^24,25^. The surface characteristics and affinity of the adsorbent are expressed by specific constant values that define the adsorption isotherm. These constant values can also be used to compare the adsorptive capacities of the adsorbent for various pollutants^26^. To identify a good model that can be applied to the design process, the current data was accurately fitted into a variety of isotherm models. The data presented in Table 1 with normalized deviation is represented by a straight line according to the applications of the Freundlich, Langmuir, BET, and Tempkin models.

**Table 1:** Comparison of the coefficients isotherm parameters for studied dyes adsorption onto nano-biosorbent.

	Congo red	Xylenol orange
Freundlich model $qe = {K}_{f}{\left.\left({C}_{e}\right.\right)}^{1/n}$		
K_f_	1.249	1.057
1/n	0.6676	0.2633
r	0.80	0.47
n	1.50	3.80
Langmuir model $qe=\frac{{Q}^{0}b{C}_{e}}{1+b{C}_{e}}$		
* Q0*	0.3099	− 8.40
* b*	0.3936	0.9458
* r*	0.84	0.78
* R*_*L*_	0.323	0.033
BET model $\frac{\frac{{C}_{e}}{{C}_{0}}}{{q}_{e}\left(1-\frac{{C}_{e}}{{C}_{0}}\right)}=\frac{1}{{K}_{b}{q}_{m}}+ \frac{{K}_{b}-1{C}_{0}}{{K}_{b}{q}_{m}{C}_{0}}$		
* Kb*	− 0.2365	− 28.78
* Qm*	2.2045	43.89
* r*	0.46	0.42
Tempkin model ${q}_{e}={B}_{T}{\text{ln}}{A}_{T+ }{B}_{T }ln {C}_{e}$		
* A*_*T*_	7.134	4.216
* B*_*T*_	0.790	0.465
* b*_*T*_	263.10	446.98
* r*	0.78	0.43

**Figure 6:**
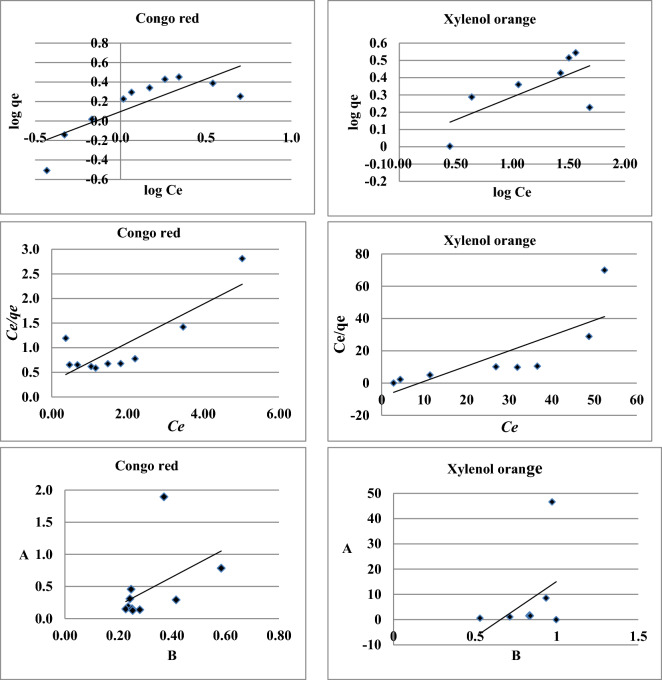
Freundlich, Langmuir and BET adsorption isotherms for Congo red and Xylenol orange with the nano-biosorbent.

**Figure 7:**
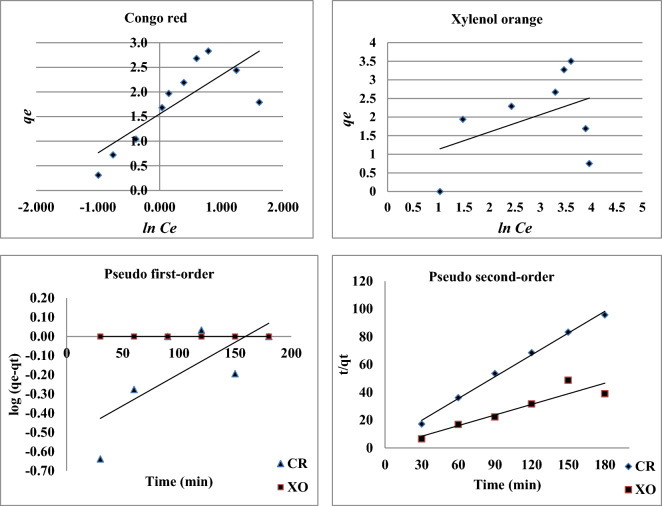
Tempkin adsorption isotherms and Pseudo first-order and second- order kinetics for studied dyes adsorption onto nano-biosorbent.

For the nano-sorbent, the isotherms that were obtained with these parameters are shown in Figs. 6 and 7, along with the experimental data points.

The relationship between the concentrations of CR and XO at equilibrium (Ce) and the amounts of dyes adsorbed per unit mass of the adsorbent (qe) is represented by the Freundlich adsorption isotherm. The Freundlich isotherm equation, represented by Eq. (2), can be used to characterize heterogeneous systems and is applicable to adsorption on heterogeneous surfaces involving interactions between adsorbed molecules.

2 $$\mathrm{qe }= {{\text{K}}}_{{\text{f}}}{\left.\left({{\text{C}}}_{{\text{e}}}\right.\right)}^{1/{\text{n}}}$$

where K_f_ is the Freundlich constant indicative of the relative adsorption capacity of the adsorbent related to the bonding energy and can be defined as the adsorption or distribution coefficient.

If n = 1, then adsorption is linear; if n < 1, then adsorption is chemical; and if n > 1, then adsorption is a favorable physical process^27^. The constant n is the heterogeneity factor that represents the deviation from linearity of adsorption. The findings show that the Freundlich isotherm equation more accurately represents the adsorption of CR on the nano-biosorbent under ideal circumstances at room temperature (25 °C) than does XO. In the case of CR, the sorption equilibrium data fit the Freundlich equations with values of 0.80 for the correlation coefficient (r), a measure of goodness of fit and n > 1. This suggests that the adsorption of the two dyes onto nano-biosorbent is a physically advantageous process. The Langmuir theory's fundamental premise is that sorption occurs at particular homogeneous energy sites in the sorbent. The next assumption, which is stated as follows in Eq. (3), is that no more sorption can occur at a site once a pollutant ion occupies it.

3 $${\text{qe}}=\frac{{{\text{Q}}}^{0}{{\text{bC}}}_{{\text{e}}}}{1+{{\text{bC}}}_{{\text{e}}}}$$

where qe is the amount adsorbed at equilibrium (in mg/g), Ce is the equilibrium concentration of the adsorbate (in mg/L), and the parameters Q^0^ and b are Langmuir constants related to maximum adsorption capacity (monolayer capacity) and bonding energy of adsorption, respectively, which are functions from the characteristics of the system as well as time^28^.

The intercept and slope of the linear plot of Ce/qe versus Ce (Fig. 6) can be used to calculate Q^0^ and b. using the Langmuir b parameter, the dimensionless constant known as the separation factor (R_L_) was computed from the following Eq. (4):

4 $${{\text{R}}}_{{\text{L}}}=\frac{1}{1+{{\text{bC}}}_{0}}$$

where b is the Langmuir constant and C_0_ is the initial concentration. Depending on the value of RL, an isotherm can be regarded as unfavorable, linear, favorable, or irreversible. The results showed that RL is 0.323 for CR and 0.033 for XO. It is thought that the adsorption of dyes onto the nano-biosorbent is advantageous because all of these RL values fall between 0 and 1.

The Brauner–Emmet–Teller (BET) isotherm, which can be expressed as follows in linear Eq. (5), was proposed to enhance the fit provided by the Freundlich or Langmuir equation:

5 $$\frac{\frac{{{\text{C}}}_{{\text{e}}}}{{{\text{C}}}_{0}}}{{{\text{q}}}_{{\text{e}}}\left(1-\frac{{{\text{C}}}_{{\text{e}}}}{{{\text{C}}}_{0}}\right)}=\frac{1}{{{\text{K}}}_{{\text{b}}}{{\text{q}}}_{{\text{m}}}}+ \frac{{{\text{K}}}_{{\text{b}}}-1{{\text{C}}}_{0}}{{{\text{K}}}_{{\text{b}}}{{\text{q}}}_{{\text{m}}}{{\text{C}}}_{0}}$$

where K_b_ is the constant that expresses the energy of interaction with the surface. BET constants, K_b_, and qm values can be calculated from the plot between $\frac{\frac{{{\text{C}}}_{{\text{e}}}}{{{\text{C}}}_{0}}}{{{\text{q}}}_{{\text{e}}}\left(1-\frac{{{\text{C}}}_{{\text{e}}}}{{{\text{C}}}_{0}}\right)}$ versus Ce/C_0_ (Fig. 6).

The findings show that the dyes' adsorption on the employed adsorbent is not the linear model that best fits either CR or XO. The Tempkin isotherm model postulates that adsorption is a uniform distribution of maximum binding energy and that the adsorption heat of all molecules in the layer decreases linearly with coverage because of adsorbate–adsorbate repulsions^29^. Tempkin equation has commonly been written in the following Eq. (6) ^30^:

6 $${{\text{q}}}_{{\text{e}}}={{\text{B}}}_{{\text{T}}}{{\text{lnA}}}_{{\text{T}}+ }{{\text{B}}}_{\mathrm{T }}\mathrm{ln }{{\text{C}}}_{{\text{e}}}$$

The equilibrium binding constant (l min^−1^) that corresponds to the maximum binding energy is known as A_T_, and it is correlated with the heat of adsorption (b_T_)^31^. The plot of qe versus ln Ce in Fig. 6 can be used to calculate the values of the isotherm constants A_T_ and b_T_. The values of the constants and correlation coefficients are given in Table 1. In contrast to Xylenol orange, which indicates that adsorbents weren't favorable to the Tempkin model, the data from the Table demonstrated that the adsorption of Congo red also followed the Tempkin model.

Therefore, the data reported in Table 1 reveal that most of the isotherm models tested have been fitted well with the experimental data obtained for the adsorption of CR dye, only BET isotherm showed less agreement with the experimental data obtained. On the other hand, most of the isotherm models tested weren’t favorable for XO unless Langmuir isotherm.

### Kinetics of dyes biosorption

Selecting the ideal operating conditions for design purposes requires consideration of the adsorbate uptake kinetics. Two widely used kinetic models, the pseudo-first-order kinetic model and the pseudo-second-order kinetic model, were used to analyze the kinetics data obtained from the adsorption of the dyes Xylenol orange and Congo red onto modified nano-biosorbent. The linear regression correlation coefficient (R^2^), a gauge of how well the predicted values from a forecast model match the experimental data, was used to choose the best fit model.

#### Pseudo-first order kinetic model

The rate of sorption site occupation is assumed to be proportionate to the number of unoccupied sites in the pseudo-first-order kinetic model. The earliest known equation describing the adsorption rate based on the adsorption capacity is the pseudo-first-order model by Lagergren ^32^. The differential equation is often written in Eq. (7) as follows:

7 $${\text{log}}({{\text{q}}}_{{\text{e}}}-{{\text{q}}}_{{\text{t}}})={{\text{logq}}}_{{\text{e}}}-\frac{{{\text{k}}}_{1}}{2.303}{\text{t}}$$

where qe and qt refer to the number of studied dyes adsorbed (mg/g) at equilibrium and at any time, t (min), respectively, and k_1_ is the equilibrium rate constant of pseudo-first-order adsorption (l min^−1^). Plotting log (qe-qt) versus t yielded values for the rate constant, k_1_, equilibrium adsorption capacity, qe, and correlation coefficient, R_2_. (Fig. 7).

Table 2 displays that our computed values of qe were less than the corresponding experimental data obtained. This suggests that the dyes under study do not always adsorb onto nano-biosorbent in a perfect pseudo-first-order reaction.

**Table 2:** Comparison of the first- and second-order adsorption rate constants and calculated and experimental *q*e values for Congo red and Xylenol dyes and nano-biosorbent.

Dyes	*q*_*e*_ (exp)	First-order kinetic model			Second-order kinetic model			
		*qe* (calc)	*k*_*1*_ × 10^3^	*R*^2^	*q*_*e*_ (calc)	*k*_*2*_ × 10^3^	*h*	*R*^2^
Congo red	1.77	− 0.526	3.30	0.526	0.523	4193	1.147	0.995
Xylenol orange	2.01	0.049	− 1.10	0.154	0.255	776	0.051	0.865

#### Pseudo-second order kinetic model

The premise behind pseudo-second order is that the rate-limiting step results from chemical sorption, which involves valence forces through the sharing or exchange of electrons between the dye ions and the adsorbent. The pseudo-second-order equation (Eq. 8) was created by rearranging the equation provided by^33^ into a linear form.

8 $$\left(\frac{{\text{t}}}{{{\text{q}}}_{{\text{t}}}}\right)=\frac{1}{{{\text{K}}}_{2}{{\text{q}}}_{{\text{e}}}^{2}}+ \frac{1}{{{\text{q}}}_{{\text{e}}}}\left({\text{t}}\right)$$

The initial adsorption rate, h (mg g^−1^ min^−1^) is expressed by the following Eq. (9):

9 $${\text{h}}={{\text{K}}}_{2}{{\text{q}}}_{{\text{e}}}^{2}$$

where k_2_ (g mg^−1^ min^−1^) is the equilibrium rate constant of pseudo-second-order adsorption. For the adsorption of CR and XO on nano-biosorbent, the pseudo-second-order kinetic model and the experimental data agree well, as seen by the linear plot of t/qt versus t (Fig. 7). The intercept and slope of the plots of t/qt versus t were used to calculate the equilibrium adsorption capacity, qe, and the second order rate constant, k_2_. The computed qe values and the experimental data (Table 2) agree extremely well. Table 2 shows that the pseudo-second-order model with good linearization (R_2_ = 0.995 and 0.865) more accurately describes the sorption kinetics of CR and XO on the modified nano-biosorbent. These findings provide more evidence in favor of the chemisorption theory explaining the sorption. Previous reports of similar results were also made by^34,35^.

## Comparison with other studies

The amount of maximum removal capacity of Congo red and Xylenol orange dye by modified Fe_3_O_4_ nano-biosorbent was 97% and 47% respectively (Table 3). This amount has been compared with removal percentage achieved from the other studies for the removal of Congo red and Xylenol orange. Considering that^40^ and^41^'s work focused on wastewater and not aqueous solution, the removal capacity of Congo red by the nano-biosorbent in the present study is essentially in line with their findings. For the same reason, the current study is focused on wastewater, the removal capability of Xylenol orange for the nano-biosorbent is lower than it was in earlier studies, and they depend on the aqueous solution.

**Table 3:** Comparison of the removal percentage for CR and XO by different adsorbents.

Dye	Adsorbent	Solution	% removal	Adsorption capacity mg/g	Reference
Congo red	*Surjana* seed powder, maize seed powder, chitosan	Aqueous solution	98.0 94.5 89.4	–	^36^
Congo red	Valoria bryopsis	Aqueous solution	97.7	0.977	^37^
Congo red	Dried roots of *Eichhornia crassipes*	Aqueous solution	96.0	1.58	^38^
Congo red	Waste black cardamom peels	Aqueous solution	96.2	–	^39^
Congo red	Modified commercial zeolite catalyst	wastewater	99.2	21.11	^40^
Congo red	Untreated sawdust	Wastewater	94.4	–	^41^
Congo Red	Activated carbon (laboratory grade)	Aqueous solution	–	1.88	^42^
Congo red	*Phragmites australis*	wastewater	97.0	0.96–3.36	Present study
Xylenol orange	Sodium dodecyl sulfate (SDS) self-microemulsifying systems (SMES)	Aqueous solution	89.7	–	^43^
Xylenol orange	Bauxite	Aqueous solution	75.0	–	^44^
Xylenol orange	Coal ash	Aqueous solution	80.0	0.74	^45^
Xylenol orange	Molecularly imprinting polymers (MIP-R2)	Aqueous solution	80.0	–	^46^
Xylenol orange	Poly urethane foam	Wastewater	99.54	0.904	^47^
Xylenol orange	Silica nanoparticles	Aqueous solution	–	9.08	^48^
Xylenol orange	*Phragmites australis*	Wastewater	47	0.18–17.58	Present study

The maximal adsorption capacity of CR and XO dye by Phragmites australis, a nano-microporous biosorbent, was found to be between 0.96 and 3.36 mg/g for CR and between 0.18 and 17.58 mg/g for XO. This amount has been contrasted with the adsorption capacity for the elimination of CR and XO obtained from other studies. Table 3 shows the maximum adsorption capacity by various sorbents. As a nano-microporous biosorbent, Phragmites australis has a maximum CR adsorption capacity that is notably greater than the maximum capacity of other sorbents. With the exception of the modified commercial zeolite catalyst by^40^, which has an adsorption capacity of 21.11 mg/g.

It is shown that *Phragmites australis*, a nano-microporous biosorbent, has a maximum XO adsorption capacity that is significantly more than the maximum capacity of other sorbents (Table 3).

## Conclusion

The efficacy of a modified nano-biosorbent derived from the manufacture of nano magnetite (Fe_3_O_4_) nano-sorbents was examined in relation to its ability to remove dyes such as Xylenol orange and Congo red from industrial wastewater by means of adsorption on the surface of macrophytes (*Phragmites australis*). A surface appropriate for adsorption was found to be uneven and porous using SEM investigation. The trials were conducted in order to generate baseline data on beginning concentrations, adsorption dye equilibrium times, ideal adsorbent dosages, and solution pH. The sorption kinetics of Xylenol orange and Congo red were found to support the pseudo-second-order model, which better reflects the sorption kinetics of the dyes under study on the modified nano-biosorbent through good linearization. Finding an appropriate model that can be used for the design process required finding an accurate fit for the current data into several isotherm models. Using the Freundlich, Langmuir, BET, and Tempkin models, the data is represented by a normalized deviation and a straight line. The data shows that, for the adsorption of Congo red dye, the majority of the isotherm models that were tested fit the experimental data with good accuracy; the BET isotherm, on the other hand, showed less agreement with the obtained experimental data. However, aside from the Langmuir isotherm, the majority of the isotherm models that were tested weren't good for Xylenol orange. Given that *Phragmites australis*, the raw material for biomass, is readily available in most wetlands as macrophytes in large quantities, the treatment approach appears to be low-cost, low-tech, and economical. Therefore, it is advised to use nanomagnetic biosorbent as an inexpensive and efficient adsorbent to remove organic dyes from industrial effluents.

## Data Availability

All data generated or analyzed during this study are included in this published article.
