# Author Correction: Utilization of aquatic biomass as biosorbent for sustainable production of high surface area, nano- microporous, for removing two dyes from wastewater

**DOI:** 10.1038/s41598-024-63256-9

**Published:** 2024-06-06

**Authors:** Maha Ahmed Mohamed Abdallah, Ahmed E. Alprol

**Affiliations:** National Institute of Oceanography and Fisheries, NIOF, Cairo, Egypt

Correction to:* Scientific Reports* 10.1038/s41598-024-54539-2, published online 23 February 2024

The original version of this Article contained errors in the Affiliation, which was incorrectly given as ‘National Institute of Oceanography and Fisheries, NIOF, Alexandria, Egypt’. The correct affiliation is listed below.

National Institute of Oceanography and Fisheries, NIOF, Cairo, Egypt.

Additionally, the original version of this Article contained an error in the spelling of the author Ahmed E. Alprol which was incorrectly given as Ahmed Eid Alprol.

The original Article has been corrected.

